# Reduction of galactose side chains in type II arabinogalactan alters homogalacturonan methyl esterification in *Arabidopsis thaliana* seed coat mucilage

**DOI:** 10.1007/s00425-025-04717-x

**Published:** 2025-05-24

**Authors:** Lucía Albornos, Paula Iriondo-Ocampo, Berta Dopico, Ignacio Martín

**Affiliations:** Departamento de Botánica y Fisiología Vegetal, Instituto de Investigación en Agrobiotecnología (CIALE), University of Salamanca, 37007 Salamanca, Spain

**Keywords:** β-galactosidase, Arabidopsis, Arabinogalactan proteins, Cell Wall, Seed coat mucilage, Type II arabinogalactan

## Abstract

**Main conclusion:**

Trimming of β-(1,3) and β-(1,6)-galactosyl residues from type II arabinogalactan side chains causes an increase in the methyl esterification degree of homogalacturonan in Arabidopsis seed coat mucilage.

**Abstract:**

Arabinogalactan proteins (AGPs) are involved in various physiological processes, such as cell elongation, xylem differentiation, resistance to abiotic stresses or secretion and adherence of seed coat mucilage, a structure suggested as a model system for cell wall studies. The specific roles of AGPs are not fully established, although their carbohydrate motif (type II arabinogalactan, AGII) seems to be essential, being able to mediate interactions with different signalling molecules or with other cell wall polysaccharides. The aim of the present work is to determine the role of AGII from AGPs in the structural organization of the cell wall, using *Arabidopsis thaliana* plants that overproduce β-galactosidase βV-Gal from *Cicer arietinum* (35S::βV-Gal plants), an enzyme that acts specifically on the β-(1,3) and β-(1,6)-galactosyl bonds of AGII. The characterization of the seed coat mucilage has allowed us to establish a cell wall homeostasis mechanism in which the neutral side chains of the AGII of the AGPs determine the degree of HG methyl esterification. Thus, the reduction in the galactose is accompanied by an increase in the level of esterification, probably as a compensatory mechanism to maintain the mechanical properties of this specialized cell wall and its hydration properties.

**Supplementary Information:**

The online version contains supplementary material available at 10.1007/s00425-025-04717-x.

## Introduction

The plant cell wall, an extracellular matrix formed by a complex network of polymers, plays a fundamental role in different aspects of growth, differentiation, and interactions with the environment and other organisms (Cosgrove 2005; Houston et al. 2016). The polysaccharide component of the cell wall consists of a network of cellulose microfibrils cross-linked by various hemicelluloses (xyloglucan, xylans and mannans) and embedded in a matrix of pectic polysaccharides. The main pectins in plant cell walls are homogalacturonan (HG), rhamnogalacturonan I (RGI) and the substituted galacturonans rhamnogalacturonan II (RGII) and xylogalacturonan (XGA) (Carpita and Gibeaut 1993; Harholt et al. 2010; Mohnen 2008). Although pectin content varies depending on the environment, tissue, and species (Zablackis et al. 1995), these polysaccharides make up approximately 35% of the primary cell wall in dicotyledonous and non-graminaceous monocots cell walls, 2–10% of primary walls in grasses and other commelinid plants, and up to 5% of walls in woody tissue (O’Neill et al. 1990; Ridley et al. 2001; Mohnen 2008). The proportion of the different pectins is also variable, but typically HG is the most abundant, constituting about 65% of the total pectic content, while RGI constitutes 20–35% (Mohnen 2008) and XGA and RGII are minor components, each comprising less than 10% (Zandleven et al. 2007; Mohnen 2008). HG is composed of linear chains of α-(1,4)-galacturonic acid, whose carboxyl groups may be methyl esterified. Unesterified regions can cross-link via Ca^2+^ bridges to form gels, thereby controlling cell wall porosity, which is also influenced by RGI branching (Willats et al. 1999; 2001a, b). These branches can be formed by neutral side chains (galactans, arabinans, and arabinogalactans) or by the HG chains themselves (Carpita and Gibeaut 1993; Vincken et al. 2003; Hongo et al. 2012).

It is apparent from previous studies that the polysaccharide component is crucial in controlling cell wall structure, although proteins such as those acting on polysaccharides, proteases, oxido-reductases, lipid-related proteins, and especially those traditionally considered as structural proteins are also relevant (Jamet et al. 2006). The latter, namely extensins or HRGPs (hydroxyproline rich glycoproteins), PRPs (proline-rich proteins) and GRPs (glycine-rich proteins) (McNeil et al. 1984; Carpita and Gibeaut 1993), also play a fundamental role in maintaining cell wall architecture (Jamet et al. 2006). Additionally, subsequent studies indicate that also arabinogalactan proteins (AGPs) have an increasingly significant function regulating the structure of the cell wall, its synthesis and deposition, although their specific role has not been fully determined (Tan et al. 2013; Zhong et al. 2011; Lin et al. 2022).

AGPs conform a complex and highly diverse family of glycosylated proteins present in all plants (Gaspar et al. 2001). These proteins are found in the apoplastic space, but also on the outer side of the plasma membrane and in secretions, such as stigma surface and wound exudates (Ellis et al. 2010; Ma and Johnson 2023). The protein skeleton is very variable, although repetitions of Ala-Pro, Ser-Pro, Thr-Pro and Val-Pro dipeptides (in which the Pro is usually hydroxylated) are frequent (Schultz et al. 2000). The carbohydrate motif of the AGPs, which can constitute up to 90% of their molecular weight, is comprised of type II arabinogalactan (AGII) molecules linked by an O-glycosidic bond to the hydroxyproline residues of the protein skeleton (Showalter 2001). AGII has a central backbone consisting of a chain of β-(1,3)-D-galactose residues with abundant β-(1,6)-D-galactose branches, which can be substituted with α-(1,3)-L-arabinose residues (Ridley et al. 2001). The size of the AGII molecule varies between AGPs, with estimations ranging between 30 and 150 sugar residues. Furthermore, depending on the species and tissue, it may contain L-rhamnose, L-fucose, D-glucuronic acid, D-xylose, D-mannose, D-glucose, D-galacturonic acid and D-glucosamine (Seifert 2020) (Leszczuk et al. 2023).

AGPs appear to be involved in various physiological processes, which is consistent with the heterogeneous nature of this protein family. Thus, they have been associated with different aspects of root biology (Hromadová et al. 2021), such as cell division, cell expansion and cell wall deposition (Serpe and Nothnagel 1994; van Hengel and Roberts 2003; Tucker et al. 2018, Seifert 2021), trichoblast definition and root hair growth (Kirchner et al. 2018; Borassi et al. 2020) and have also been reported as components of root exudates and cell walls of root cap cells (Driouich et al. 2019; Galloway et al. 2020). They also participate in the differentiation of xylem vessels and secondary cell wall deposition (Motose et al. 2004; Zhang et al. 2022), the resistance to abiotic stresses (Lee et al. 2008; Hromadová et al. 2021), and in the responses of plants to beneficial or pathogenic microorganisms (Villa-Rivera et al. 2021). Moreover, AGPs are particularly involved in the biology of reproduction, being determining factors during the processes of female gametogenesis (Acosta-Garcia and Vielle-Calzada 2004; Moreira et al. 2022), male gametophyte development and pollen tube growth (Mollet et al. 2002; Lee et al. 2008; Coimbra et al. 2009; Lopes et al. 2019). Also, these proteins have been related to fruit metabolism and have been proposed to participate in fruit ripening an abscission (Perrakis et al. 2019; Leszczuk et al. 2020). In addition, AGPs seem to be essential for the correct formation of the seed mucilage present in various species (including *Arabidopsis thaliana*), being determinant in both, its secretion and its adhesion to the seed coat (Griffiths et al. 2014; Ajayi et al. 2021; Kaur et al. 2021). Despite being related to all these processes, AGPs specific role is not fully established. Nevertheless, it seems clear that the carbohydrate motif plays a determining role in their function, being able to mediate their interaction with different signalling molecules or with other cell wall polysaccharides (Seifert 2020), as some authors have proposed in the case of seed coat mucilage (Griffiths et al. 2014).

During their formation, the seeds of *A. thaliana* accumulate structural polysaccharides in the apoplast of the epidermal cells. When the seed is rehydrated, they are released and form a mucilaginous cover rich in pectins that surrounds the seed, protecting it and helping to maintain its hydration (Macquet et al. 2007). The composition of this mucilage is very similar to that of the primary cell wall, hence the cell wall research community consider it a suitable structural model, as it is amenable to functional genetics, allows for the visualization and quantification of cell wall components and for the study of their synthesis, secretion and assembly, among other approaches (Arsovski et al. 2010).

The mucilage-secreting cells of the seed coat differentiate from the outermost cell layer of the outer integument of the ovule, which after fertilization begin to synthesize mucilage and secrete it to the apoplast between the primary wall and the plasma membrane. As this occurs, a central cytoplasmic column is created and gradually replaced by a cellulose-rich secondary cell wall-like structure called columella that mediates mucilage arrangement and retention (Stork et al. 2010; Mendu et al. 2011). Once the seed is hydrated, the mucilage expands, pressing the primary cell wall and being released into the medium. After the mucilage has been hydrated and released, two layers can be distinguished. The outer one is a cloud-like, diffuse and easily extracted with water structure, which is known as non-adherent mucilage, and is mostly composed of RGI (Macquet et al. 2007). The most inner one, known as adherent mucilage, is in direct contact with seed coat, and is made up of RGI (the main component), but also contains considerably amounts of cellulose, HG, and arabinan and galactan-side chains (Macquet et al. 2007).

Various proteins are involved in the mucilage metabolism and its structural organization, including AGPs (Arsovski et al. 2010). Multiple studies have shown that AGPs, despite not being a major component of mucilage, are determinant for its secretion, expansion and for their adhesion to seed coats (Griffiths et al. 2014; Ajayi et al. 2021). The mechanism by which AGPs influence the structure of mucilage is unknown, but it is believed that they could be interacting with the rest of the pectic components or even influencing their interaction with the cellulose fibers of the columella (Kaur et al. 2021).

In previous works, we have tried to deepen in the role of various β-galactosidases from chickpea (*Cicer arietinum*) and *A. thaliana* in cell wall metabolism (Esteban et al; 2005; Albornos et al. 2012; Moneo-Sanchez et al. 2018). The results of these studies pointed to a possible action of chickpea β-galactosidase V (βV-Gal, encoded by *CarBGal5*) on the neutral galactose side chains of AGII (Martín et al. 2009), something that we have confirmed in the present work. Once the substrate specificity was established, we constructed *A. thaliana* plants overproducing chickpea βV-Gal under the control of the p35S promoter of the cauliflower mosaic virus (35S::βV-Gal plants). Our aim was to generate a tool to study the role of AGII, and more specifically of its galactosyl residues, in the structural organization of the cell wall by trimming β-(1,3) and β-(1,6) galactose side chains from AGII *in muro*. Thus, the main objective of this work is to characterize the changes induced by βV-Gal in arabidopsis seed mucilage to shed light in the function of galactose substitutions of AGII (and by extension of AGPs) in the correct organization of this specialised seed tissue. Eventually, considering the suitability of mucilage as a model system for cell wall studies, our results will set the basis to deepen in the function of AGII in the structural organization of the cell walls throughout plant growth and development.

## Materials and methods

### Plant material and growth conditions

*Arabidopsis thaliana* Columbia (Col) ecotype was used as wild-type (WT) background for genetic transformation. Seeds from WT and transgenic lines were surface sterilized as described by Albornos et al. (2012) and cold treated at 4 °C for 3 days before sowing. Seeds were grown in Petri dishes on one-half-strength Murashige and Skoog (1962) agar medium with 1% (w/v) sucrose. Plates were maintained in a growth chamber (Aralab, Rio de Mouro- Portugal) at 22 °C with both a 16 h photoperiod (provided by cool white fluorescent tubes, an irradiance of approximately 80–100 μmol m^−2^ s^−1^), or in darkness to obtain etiolated seedlings. To obtain adult plants, 10-day-old green seedlings were transferred to plastic pots containing a 3:1 mixture of potting soil and Vermiculite and grown under the same conditions. Seeds were collected after complete senescence of adult plants.

For agroinfiltration experiments, *Nicotiana benthamiana* seeds were sown in potting soil and allowed to grow for 6 weeks in a growth chamber (Aralab) at 25 °C and 16 h photoperiod.

### Construction of expression vectors and plant transformation

*35S::CarBGal5* construct was prepared using Gateway® cloning technology (Invitrogen, Waltham, MA, USA), according to manufacturer’s instructions. *Cicer arietinum CarBGal5* cDNA, coding for βV-Gal β-galactosidase (Esteban et al. 2005), was amplified by PCR adding the attB1 and attB2 sequences at 5′- and 3′-ends, respectively. The amplified products were gel purified and used in BP reaction with pDONR201, and the entry clones generated were used in LR reaction with pK7 WG2, to allow *CarBGal5* expression under 35S promoter (Karimi et al. 2007). All constructs were verified by sequencing (primers listed on Supplementary Table S1). The generated *35S::CarBGal5* construct was electroporated into *Agrobacterium tumefaciens* strain C5851 m and *A. thaliana* plants were transformed by floral dip method (Clough and Bent 1998). Seeds harvested from infiltrated plants were screened on the appropriate antibiotic and resistant seedlings (T1) were selected. Single insertion transformant T2 plants were screened by Southern blot according to the method described by Esteban et al. (2005), using the complete *CarBGal5* ORF as probe. The expressor lines (named 35S::βV-Gal) were selected by reverse-transcription semi-quantitative PCR (RT-sqPCR) using *ACT2* as internal control (primers in Supplementary Table S1).

### Agroinfiltration of *Nicotiana benthamiana* leaves and activity assays

For heterologous expression in *N. benthamiana* leaves, *CarBGal5* ORF was PCR amplified adding the attB1 and attB2 sequences at 5′- and 3′-ends and cloned into the pDONR201 vector. The entry clones were used in LR reaction with the pEAQ-HT-DEST1 vector (Sainsbury et al. 2009; provided by Plant Bioscience Ltd, Norwich, UK). All constructs were verified by sequencing. The primers used are listed in Supplementary Table S1. The expression constructs and the GFP-containing pEAQ-GFP-HT vector used as control (also provided by Plant Bioscience Ltd., Norwich, UK) were electroporated into *A. tumefaciens* strain AGL1 and *N. benthamiana* leaves were agroinfiltrated as indicated in Izquierdo et al. (2018). Cell wall proteins were isolated from *N. benthamiana* leaves 6 days after the inoculation according to Izquierdo et al. (2018) and quantified with the Protein Assay from Bio-Rad (Hercules, CA, USA). The hydrolytic activity of the purified protein was tested using p-nitrophenyl (pNP) derivatives as substrates as described in Moneo-Sánchez et al. (2020). pNP-α-l-arabinofuranoside, pNP-α-l-fucopyranoside, pNP-α-d-galactopyranoside, pNP-β-d-galactopyranoside, pNP-α-d-glucopyranoside, pNP-β-d-glucopyranoside, pNP-β-d-mannopyranoside, and pNP-β-d-xylopyranoside, from Sigma-Aldrich were used. Activity was also assayed against lupin β-(1,4)-galactan pre-treated with α-l-arabinofuranosidase and a mix of xyloglucan oligosaccharides containing β-(1,2)-galactose (Megazyme, Wicklow, Ireland); β-(1,4)-galactobiose, larch wood arabinogalactan, and lactose (Sigma-Aldrich); β-(1,3)-galactan, β-(1,3)(1,6)-galactan, β-(1,3)-galactobiose, β-(1,3)-galactotriose, β-(1,6)-galactobiose, and β-(1,6)-galactotriose (kindly supplied by Dr. T. Kotake, Saitama University, Japan). The reaction mixtures were prepared according to Izquierdo et al. (2018). The reaction products were separated by thin-layer chromatography on silica gel plates (Merck, Darmstadt, Germany) and the galactose released was quantified in a CS-9000 dual-wavelength flying-spot scanner densitometer (Shimadzu, Kioto, Japan), using commercial galactose (Sigma-Aldrich) as standard. βV-Gal activity was estimated by subtracting the activity of leaves transformed with the GFP construct. Activities that did not show statistically significant differences between the extracts of plants transformed with the *CarBGal5* gene and the control were considered as not detected.

### Staining and immunolabelling of seed coat mucilage

Mucilage staining was performed on approximately 20 dry seeds in all cases. For general mucilage visualization, the seeds were stained with Ruthenium Red for 30 min, mounted in distilled H_2_O and visualized, with a Leica DM 4000 LED microscope (Leica Microsystems, Wetzlar, Germany) equipped with a DFC550 digital camera from the same company. To visualize only the adherent mucilage, the seeds were shaken at 250 rpm for 30 min in H_2_O and then stained with Ruthenium Red for 30 min under shaking. After staining, they were washed and visualized as indicated in the previous case, and quantified with the ImageJ 1.34S software.

For whole-seed immunolabelling seeds were previously shaken in water for 10 min and in phosphate-buffered saline (PBS) containing 5% fat-free milk powder for 1 h. The supernatant (soluble mucilage components) was removed, and the remaining seeds were immunolabelled according to Willats et al. (2001a), with slight modifications. Primary antibodies were used at a 1:10 dilution in PBS with 5% milk powder. Secondary antibodies (FITC-conjugated anti-mouse or anti-rat IgG) were also diluted (1:300) in PBS with 5% milk powder. After incubation with the secondary antibody, the sections were stained with calcofluor (0.25 mg/ml in PBS). For visualisation and imaging, the samples were mounted in Citifluor antifading reagent (Agar Scientific, Stansted Mountfitchet, UK) and a Leica SP5 confocal microscope (Leica Microsystems) was used. Fluorescence was quantified using the ImageJ 1.34S software over the maximum projections of the channel corresponding to the FITC signal.

### Extraction of mucilage from the seed coat

To extract the seed coat mucilage, a sequential fractionation was carried out according to a method modified from Huang et al. (2011) to separate the different polysaccharide fractions according to their solubility. In all cases, 50 mg of dry seeds were shaken at 250 rpm for 30 min in 500 μl of H_2_O, centrifuged for 1 min at 1000 xg and the supernatant, containing the water-soluble polysaccharides (non adherent mucilage), was collected. After the seeds were washed twice with distilled H_2_O, the adherent mucilage was sequentially extracted with 500 μl of 50 mM CDTA, pH 7.5, and 4 M KOH containing 1% w/v NaBH_4_ in the same conditions. The pH of KOH extract was neutralized with acetic acid. For ELISA assays a 1:25 dilution of each fraction in PBS was used. The amount of total sugars contained in each mucilage fraction was determined by the Anthrone method (Dreywood 1946), using glucose as standard. The uronic acid content was determined by the Filisetti-Cozzi and Carpita method (1991) using galacturonic acid as standard. In both cases, samples were fivefold diluted in H_2_O and the standard curve prepared in the same conditions.

### ELISA assays

ELISA assays were conducted according to Cornuault et al. (2014). After overnight incubation at 4 °C with the corresponding antigen, microtitre plates were washed six times with H_2_O and shaken dry. Microtitre plate wells were blocked using 200 μl per well of 5% w/v milk powder in PBS (137 mM NaCl, 2.7 mM KCl, 10 mM Na_2_HPO4, and 2 mM KH_2_PO_4_, pH 7) for 2 h at room temperature. After washing, primary antibodies were added at 1:25 dilution in 5% w/v milk powder/PBS, and incubated for 1 h. Plates were washed six times with H_2_O, shaken dry, and incubated with 100 μl per well of secondary antibody (anti-rat or anti-mouse horseradish peroxidase-conjugated IgG; Sigma-Aldrich, St. Louis, MA, USA) at 1:1000 dilution in 5% milk/PBS for 1 h at room temperature. After extensive washing in H_2_O, plates were developed using 100 μl of substrate per well (0.1 M sodium acetate buffer, pH 6, 1% tetramethyl benzidine, 0.006% H_2_O_2_). The enzyme reaction was stopped by adding 50 μl of 2.5 M H_2_SO_4_ to each well, and the absorbance read at 450 nm.

### Uronic acid methylation analysis.

The analysis of the uronic acids methylation degree was performed on the CDTA-extracted mucilage fraction. Samples were demethylated with NaOH and the released methyl groups were quantified by the alcohol oxidase method as indicated by Ajayi et al. (2021) using methanol as a standard. The uronic acid content was determined by the Filisetti-Cozzi and Carpita method (1991) and the methylation degree was calculated as the percentage molar ratio of methanol to uronic acid (Ralet et al. 2016).

### Determination of pectin methyl esterase (PME) activity.

To determine the PME activity, proteins were extracted from 200 mg of WT and 35S::βV-Gal seeds in 400 µl of extraction buffer (1 M NaCl, 12.5 mM citric acid, and 50 mM Na_2_HPO_4_, pH 6.5) as described by Saez-Aguayo et al. 2013. Equal amounts of protein (10 μg) were loaded into 6 mm diameter wells on agarose gels (1% agarose, 12.5 mM citric acid and 50 mM Na2HPO4, pH 6.5.) with 0.1% (w/v) esterified citrus pectin (Sigma-Aldrich). After incubation for 24 h at 28 °C, the plates were stained with ruthenium red (0.05%, w/v) and the halos were quantified with the ImageJ 1.34S software.

### Antibodies used

All monoclonal antibodies used in this work, along with the corresponding information on specificity and related literature, are available on CarboSource Services (http://www.carbosource.net) and Agrisera (https://www.agrisera.com) websites. Regarding type II AGPs we have used JIM14 and JIM16 antibodies, specific for three consecutive β−1,6-linked Gal units and β−1,3-linked galactan backbone substituted with a single β−1,6-linked Gal residue, respectively. Regarding the rest of cell wall polysaccharides, we have used antibodies against RGI (INRA-RU2), β-D-(1,4)-galactan (LM5), HG (non-methyl esterified: LM19, partially methyl esterified: JIM7, methyl esterified: LM20), XG (LM25), β-(1,4)-mannan oligosaccharides (LM21), xylan/heteroxylan (CCRC-M139) and glucuronoxylan (LM28). Also, to ensure the presence of the βV-Gal in the protein extracts of the transgenic seeds, previously generated Anti-βV-Gal antibodies were used (Martín et al. 2009).

## Results

### βV-Gal acts specifically on the β-(1,3) and β-(1,6)-galactosyl bonds of AGII

To establish βV-Gal substrate specificity, the enzyme was transiently produced in *Nicotiana benthamiana* leaves using the pEAQ/AGL1 agroinfiltration system described in Materials and Methods and tested against p-nitrophenyl (pNP) derivatives and different galactose containing poly- and oligosaccharides. As expected, βV-Gal was mainly active against the pNP-β-d-galactopyranoside, although trace levels of activity were detected against pNP-α-d-galacto and pNP-α-d-glucopyranoside (Table 1). Analyses against galactose-containing oligo- and polysaccharides pointed to a high specificity of βV-Gal against β-(1,3) and β-(1,6) linkages, especially against the latter, showing the highest activity when tested with β-(1,6)-galactotriose and galactobiose and gum arabic (Table 2). No activity was detected against oligosaccharides containing β-(1,2)- and β-(1,4)- linked galactose. These results point to an action of βV-Gal on the galactose side chains of type II arabinogalactan (AGII) mainly present in arabinogalactan proteins (AGPs) (Serpe and Nothnagel 1999).

**Table 1:** Substrate specificity of βV-Gal toward pNP substrates expressed as nkat/mg protein

Substrate	Activity
pNP-β-D-galactopyranoside	2.11 ± 0.06**
pNP-αD-galactopyranoside	0.81 ± 0.02**
pNP-β-D-glucopyranoside	n.d
pNP-αD-glucopyranoside	0.36 ± 0.11*
pNP-αL-arabinopyranoside	n.d
pNP-β-D-fucopyranoside	n.d
pNP-β-D-mannopyranoside	n.d
pNP-β-D-xylopyranoside	n.d

Values are the means of three biological replicates ± SD

*nd* not detected

Asterisks indicate the levels of significance (Student’s t test): *p < 0.05; **p < 0.01

**Table 2:** Substrate specificity of βV-Gal toward galactose poly- and oligosaccharides expressed as pkat/mg protein

Substrate	Activity
β-(1,4)-galactan	n.d
β-(1,3)-galactan	4.40 ± 0.45*
β-(1,3)(1,6)-galactan	28.72 ± 8.7*
Gum arabic	94.58 ± 11.36**
Arabinogalactan	2,08 ± 0,07**
XG oligosaccharides	n.d
β-(1,4)-galactobiose	n.d
Lactose	n.d
β-(1,3)-galactobiose	69.68 ± 9.19**
β-(1,3)-galactotriose	1,01 ± 0,01*
β-(1,6)-galactobiose	77.84 ± 8.06**
β-(1,6)-galactotriose	126.18 ± 0,46**

Values are the means of three biological replicates ± SD

*nd* not detected

Asterisks indicate the levels of significance (Student’s t test): *p < 0.05; **p < 0.01

### Overproduction of βV-Gal in *Arabidopsis thaliana* causes a reduction of galactose residues of AGII in seed coat mucilage

We have generated Arabidopsis plants overexpressing chickpea *CarBGal5* coding for βV-Gal β-galactosidase under 35S CaMV promoter with the aim to reduce β-(1,3) and β-(1,6) galactose side chains from AGII. After transformation and screening for antibiotic resistance over T1 seeds a high number of 35S::βV-Gal transgenic lines were identified. We selected 25 lines from T2 generation for Southern blot analyses to determine the number of transgene insertions in their genome. Four individual lines with a single insertion of the transgene were selected and screened for homozygosis.

Transgene transcript accumulation in 35S::βV-Gal selected homozygous T3 lines was determined by RT-PCR using specific primer sets for *CarBGal5* (Supplementary Table S1). Two lines with different but high levels of expression were selected for subsequent analyses: 3.13.2 and 2.3.2 (Supplementary Fig. S1), referred hereon as 35S::βV-Gal.1 and 35S::βV-Gal.2, respectively. The presence of *CarBGal5* transcripts and βV-Gal protein (using previously generated anti-βV-Gal antibodies) was also confirmed in mature seeds from 35S::βV-Gal plants (Supplementary Fig. S2a and b, respectively).

**Fig. 1:**
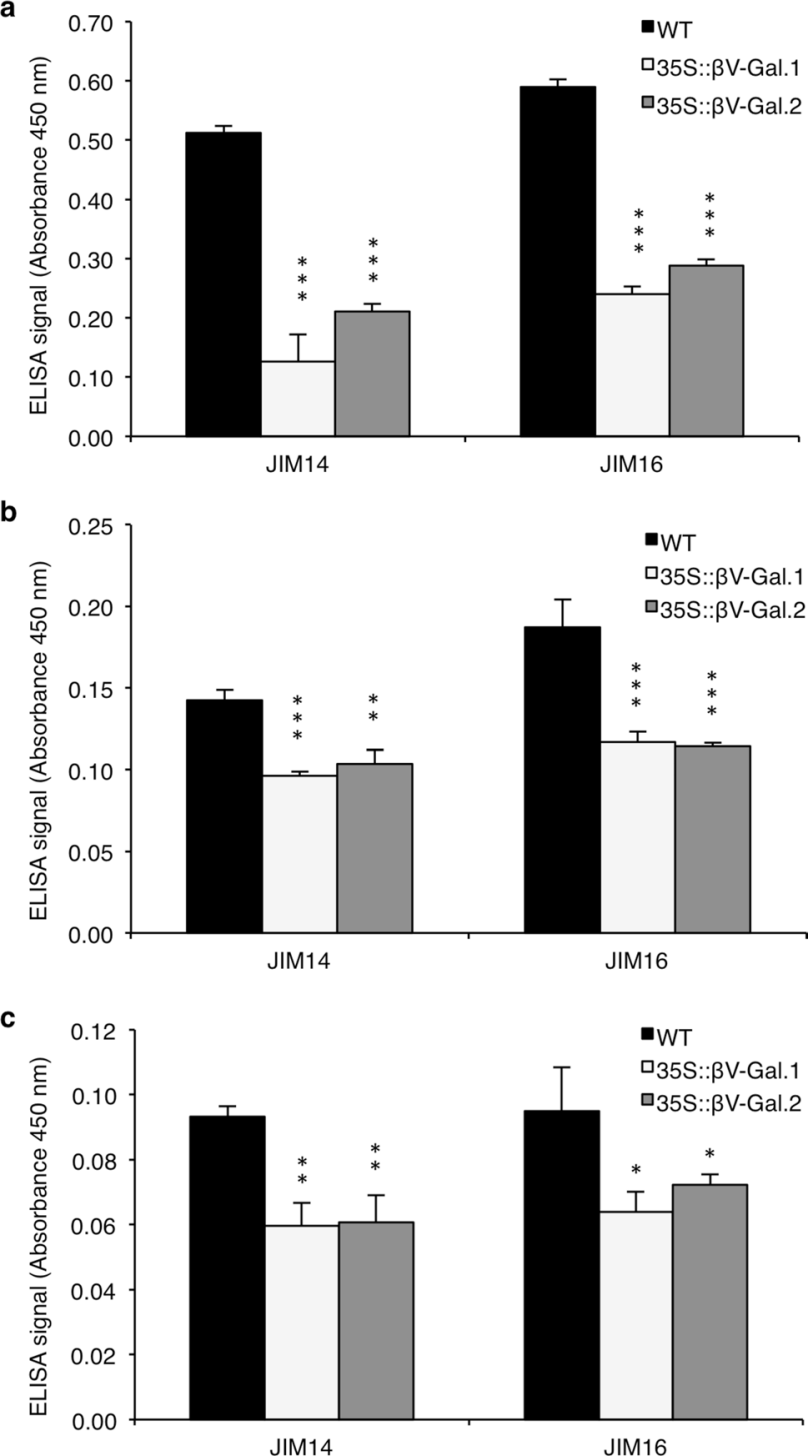
ELISA analysis using anti-AGII antibodies JIM14 and JIM16 against proteins extracts (**a**), CDTA-extracted (**b**) and KOH-extracted mucilage (**c**) from WT and transgenic 35S::βV-Gal.1 and 35S::βV-Gal.2 seeds. Values are the means of three biological replicates ± SD. Asterisks indicate the levels of significance (Student’s t test): *p < 0.05; **p < 0.01; ***p < 0.001. Signal intensity should not be compared between different antibodies as their epitope binding affinities can vary

Trimming of galactose chains of AGII in 35S::βV-Gal seeds was confirmed by ELISA experiments with JIM14 and JIM16 antibodies, specific for three consecutive β-1,6-linked Gal units and β-1,3-linked galactan backbone substituted with a single β-1,6-linked Gal residue, respectively (Ruprecht et al. 2017). Both antibodies were tested against protein extracts from whole mature WT and 35S::βV-Gal dry seeds extracted as indicated in Materials and methods (Fig. 1a) and seed coat mucilage (Fig. 1b, c). To analyse the mucilage, we carried out a sequential extraction with H_2_O (to extract the non-adherent mucilage), CDTA (pectin-enriched fraction of adherent mucilage) and KOH, to extract the polysaccharides that remain attached to the seed coat columella. Both epitopes were detected in protein extracts (Fig. 1a) and, at much lower levels, in the CDTA- and KOH-extracted adherent mucilage (Fig. 1b, c), whereas no signal was detected in the H_2_O fraction (non-adherent mucilage). As seen in this figure, the signals for both antibodies are greatly decreased in 35S::βV-Gal protein and mucilage extracts when compared to the WT, thus confirming the action of *C. arietinum* βV-Gal on galactose residues of Arabidopsis AGII.

### Trimming of galactose side chains of AGII has only minor impact in the amount of mucilage in 35S::βV-Gal seeds

**Fig. 2:**
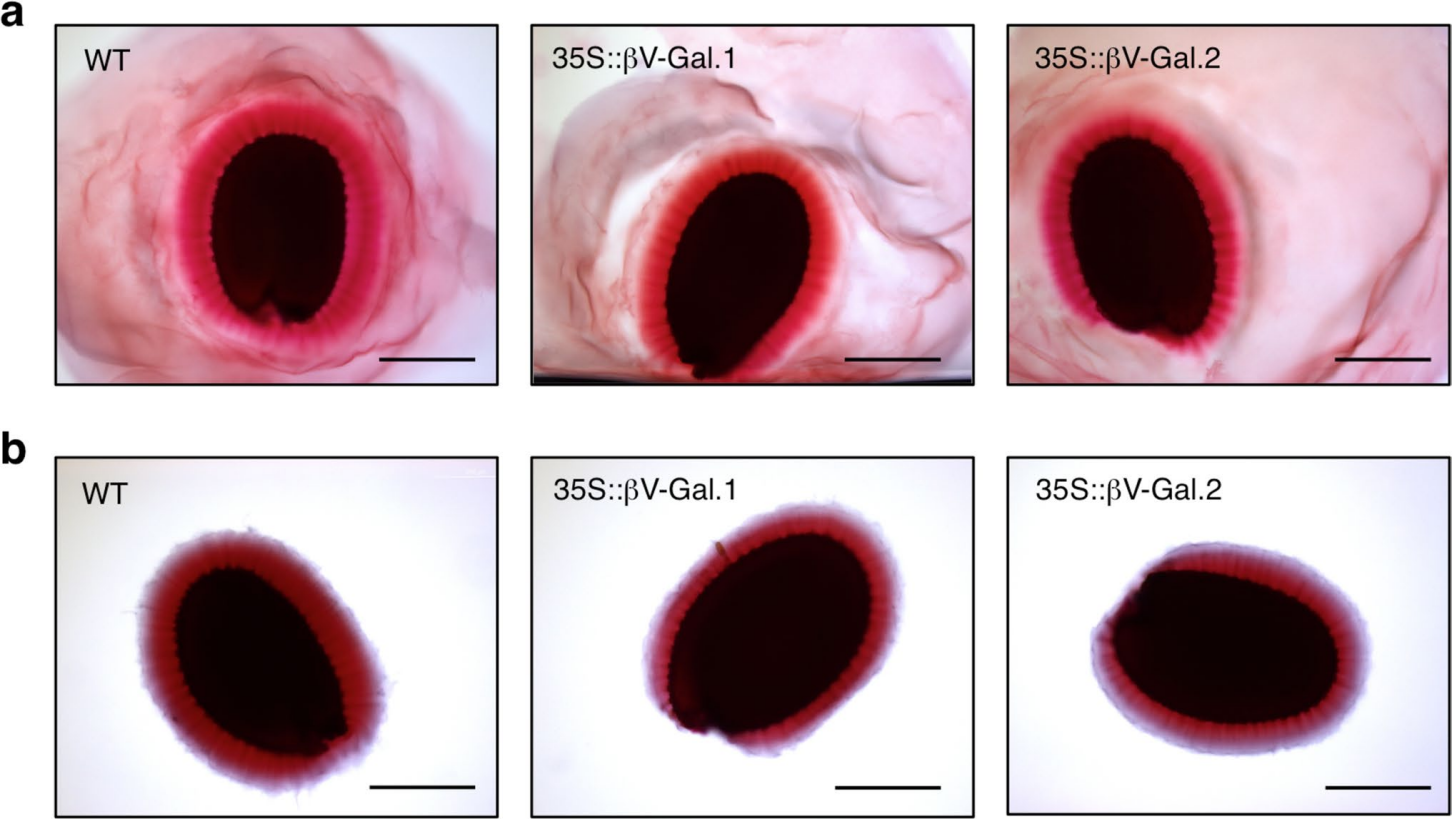
WT, 35S::βV-Gal.1 and 35S::βV-Gal.2 seeds stained with ruthenium red after being hydrated for 30 min without shaking (**a**) and after shaking in H_2_O for 30 min at 250 rpm (**b**). Scale bars = 200 µm

As a first approach for the characterization of the seed coat mucilage, we stained the seeds with ruthenium red, a dye with high affinity for the acid groups of pectins. As seen in Fig. 2a (seed hydrated for 30 min without shaking), the non-adherent mucilage shows similar intensity in WT and 35S::βV-Gal seeds. However, staining of adherent mucilage is less intense in transgenic seeds as seen both, before (Fig. 2a) and after shaking (Fig. 2b, quantified in Supplementary Fig. S3).

To check whether these changes in staining intensity corresponded to changes in the amount of mucilage, we analysed the total sugars and uronic acids content in each layer (Table 3) after the sequential extraction described above. After CDTA treatment, almost no staining was detected with ruthenium red, pointing that most pectin-enriched adherent mucilage is extracted in this step (Supplementary Fig. S4).

**Table 3:** Amount of total sugars and uronic acids present in the mucilage of WT, 35S::βV-Gal.1 and 35S::βV-Gal.2 seeds after sequential extraction with H_2_O (non adherent mucilage), CDTA and KOH (adherent mucilage)

		Non adherent mucilage (H_2_O)	Adherent mucilage (CDTA)	Adherent mucilage (KOH)
	WT	15.42 ± 0.34	3.58 ± 0.17	8.36 ± 0.17
Total sugars	35S::βV-Gal.1	13.34 ± 0.07**	3.06 ± 0.25	9.26 ± 0.69
	35S::βV-Gal.2	14.07 ± 0.19*	2.82 ± 0.6	8.87 ± 1.67
	WT	11.86 ± 0.14	2.90 ± 0.27	1.74 ± 0.06
Uronic acids	35S::βV-Gal.1	10.64 ± 1.42	2.21 ± 0.37	1.71 ± 0.67
	35S::βV-Gal.2	10.57 ± 0.85*	2.11 ± 0.33*	1.61 ± 0.42

Data are expressed as µg of sugar/mg of seed. Values are the means of three biological replicates ± SD. Asterisks indicate the levels of significance (Student’s t test): *p < 0.05; **p < 0.01

As seen in Table 3, the amount of total sugars and uronic acids is slightly lower in the seeds of the transgenic plants, both in the fraction corresponding to the non-adherent mucilage (H_2_O) and the adherent mucilage (CDTA), although only in the case of total sugars in the non-adherent mucilage the differences were statistically significant for both lines. In KOH extracts, the amount of extracted sugars is considerably lower, and no differences between transgenic lines and WT are detected (Table 3).

### Pectins from the adherent mucilage of 35S::βV-Gal seeds show higher levels of HG methyl esterification

These minor changes in the amount of polysaccharides could indicate that the differences observed in the ruthenium red staining between WT and 35S::βV-Gal seeds are due to an altered polysaccharide composition. Thus, we decided to carry out a more complete characterization of the mucilage by analysing the levels of the main pectic and hemicellulosic polysaccharides in the ELISA experiments using specific antibodies against most abundant cell wall epitopes. As indicated in the introduction, RGI has been described as the main component of the seed mucilage, although it may present small amounts of HG, neutral galactan side chains and different hemicelluloses (Macquet et al. 2007). Therefore, we decided to analyse the levels of each of these polysaccharides using monoclonals antibodies against RGI (INRA-RU2), β-D-(1,4)-galactan (LM5), HG (non-methyl esterified: LM19, partially methyl esterified: JIM7, methyl esterified: LM20), XG (LM25), β-(1,4)-mannan oligosaccharides (LM21), xylan/heteroxylan (CCRC-M139) and glucuronoxylan (LM28). It is worth mentioning that the values represented in the ELISA bar plots should not be compared between different antibodies, as they may have different affinities for their corresponding epitopes.

In accordance with the absence of changes in staining with ruthenium red in this layer of mucilage, the ELISA analysis of the pectic and hemicellulosic polysaccharides from the non-adherent mucilage (H_2_O-extrated mucilage) show no significant changes with any of the antibodies used (Supplementary Fig. S5), besides a slight non-statistically significant increase in the levels of XG recognized by LM25 antibody in 35S::βV-Gal seeds (Supplementary Fig. S5b).

**Fig. 3:**
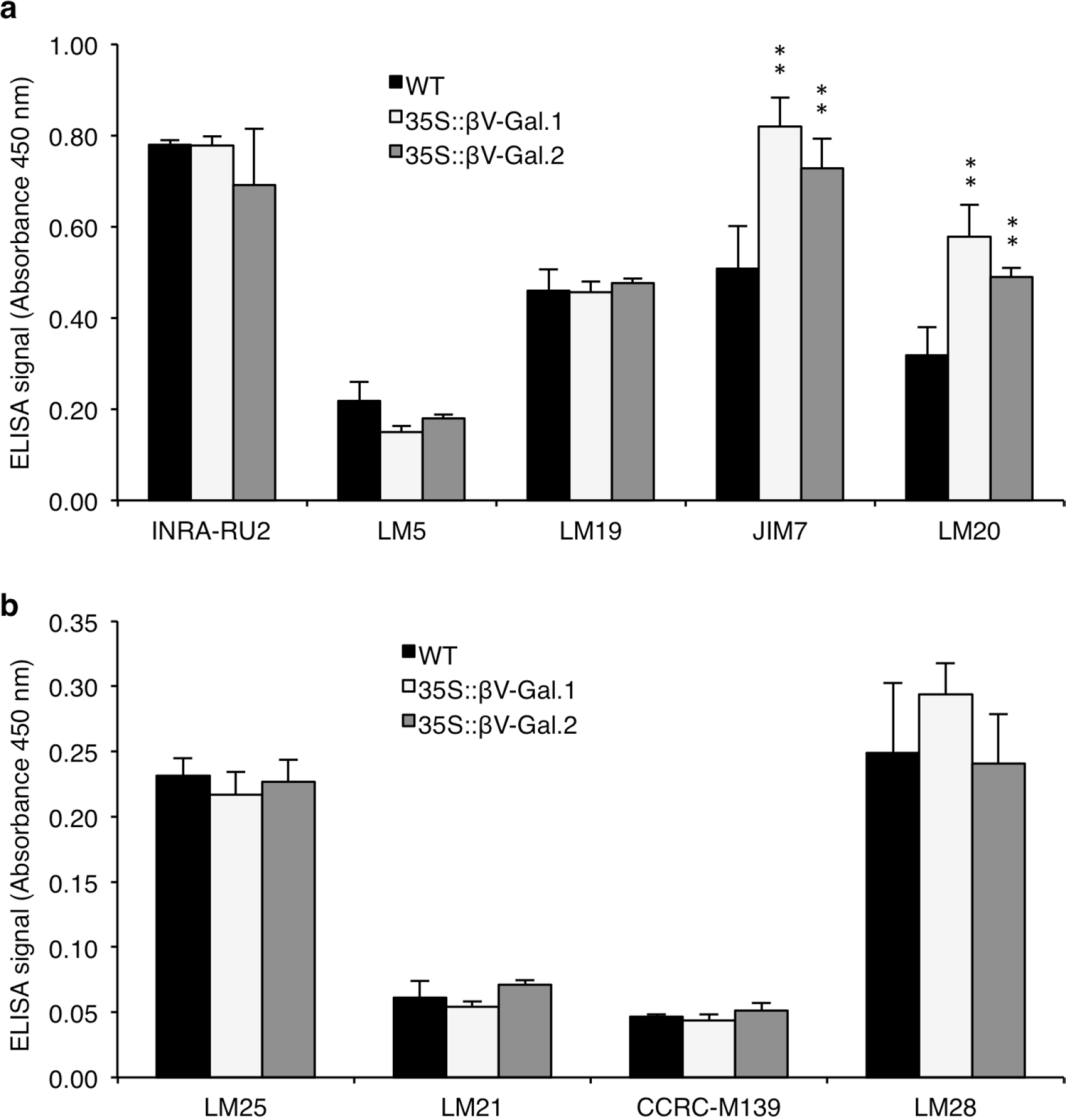
ELISA analysis of adherent mucilage extracted with CDTA from WT, 35S::βV-Gal.1 and 35S::βV-Gal.2 seeds. **a** ELISA signal for antibodies against pectic polysaccharides RGI (INRA-RU2), β-D-(1,4)-galactan (LM5) and HG (non methyl esterified: LM19, partially methyl esterified: JIM7, methyl esterified: LM20). **b** ELISA signal for antibodies against the hemicelluloses XG (LM25), β-(1,4)-mannan oligosaccharides (LM21), xylan/heteroxylan (CCRC-M139) and glucuronoxylan (LM28). Values are the means of three biological replicates ± SD. Asterisks indicate the level of significance (Student’s t test): **p < 0.01. Signal intensity should not be compared between different antibodies as their epitope binding affinities can vary

In the case of the pectins present in the CDTA-extracted adherent mucilage (Fig. 3a), methyl esterified HG recognized by JIM7 and LM20 antibodies exhibit a notable increase in 35S::βV-Gal mucilage (Fig. 3a), whereas HG recognized by LM19 (non-methyl esterified) remains unchanged. In this fraction, none of the hemicelluloses analysed show notable changes between WT and transgenic seeds (Fig. 3b), as happens in KOH extracts, where no differences are detected with any of the antibodies used between WT and 35S::βV-Gal seeds (Supplementary Fig. S6). It is worth noting that this figure does not include the signal corresponding to the JIM7 and LM20 antibodies since the high concentration of KOH used in the sequential extraction induces demethylation of HG.

**Fig. 4:**
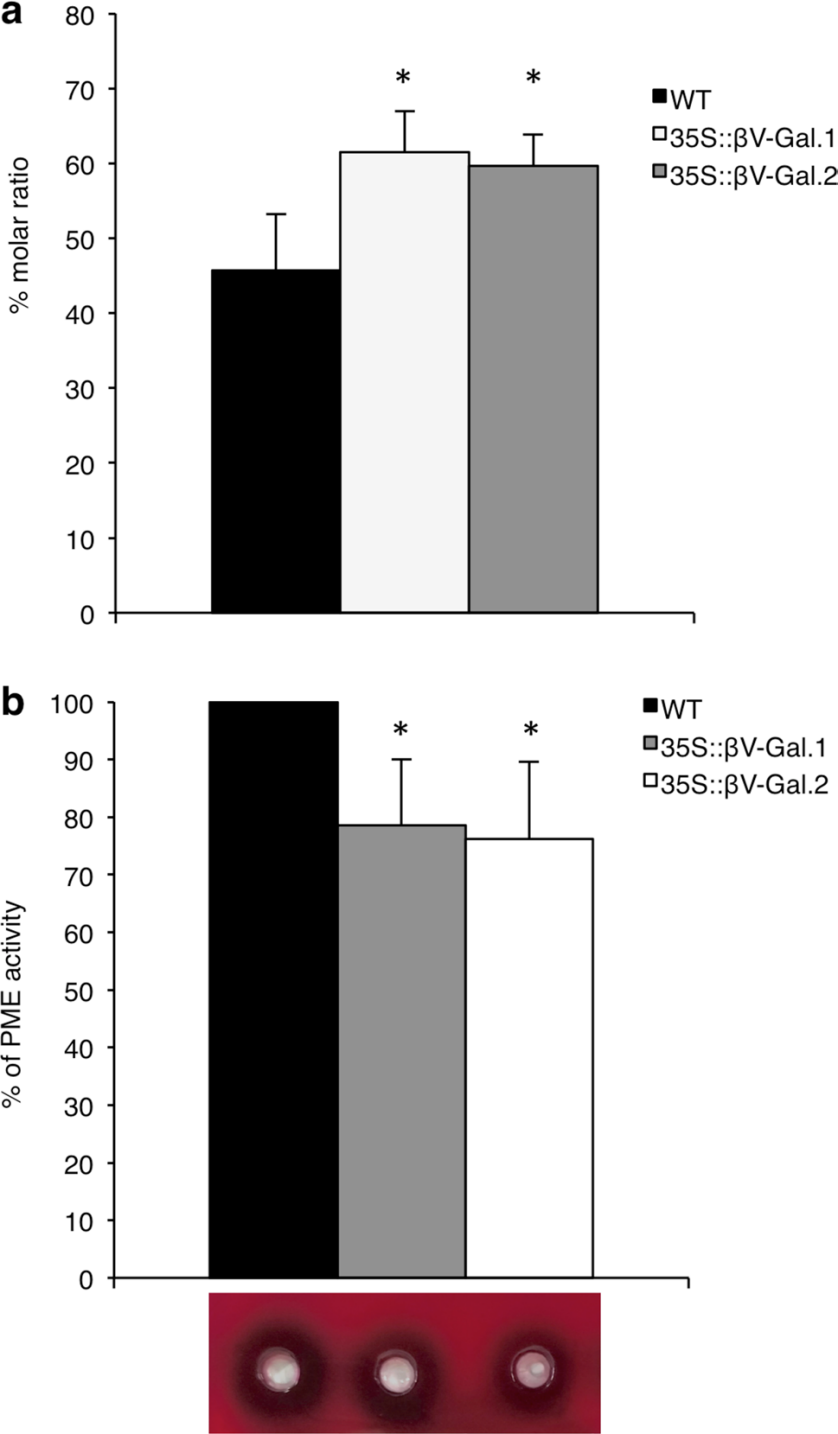
**a** Degree of pectin methyl esterification in adherent mucilage of WT, 35S::βV-Gal.1 and 35S::βV-Gal.2 seeds (data expressed as percentage molar ratio of methanol to uronic acid). **b** Pectin methyl esterase (PME) activity in protein extracts from WT, 35S::βV-Gal.1 and 35S::βV-Gal.2 seeds. Results are expressed as a percentage of variation in transgenic lines with respect to the WT, to which a value of 100% is assigned. An example of a ruthenium red-stained pectin/agarose gel showing demethylation of pectins by each protein extract is included below the bar graph. Values are the means of three biological replicates ± SD. Asterisks indicate significance level (Student’s t): *p < 0.05

This increase in the signal of the LM20 antibody in the CDTA-extracted adherent mucilage corresponds to an increase in the degree of pectin methyl esterification (Fig. 4a), which, in turn is accompanied by a decrease in PME activity in both transgenic lines (Fig. 4b). This result, along with the fact that only minor changes were detected in the levels of uronic acids between WT and transgenic seeds (Table 3), point to an increase in the methyl esterification degree of HG rather than an increase in the total amount of this pectic polysaccharide. It should be noted that PME is the only activity that shows significant variations among all those analysed in the protein extracts (in addition to the expected increase in galactosidase activity) (Fig. 4 and Supplementary Fig. S7), which is in agreement with the absence of changes in the rest of the cell wall polysaccharides, as determined in ELISA experiments (Fig. 3, S5 and S6).

**Fig. 5:**
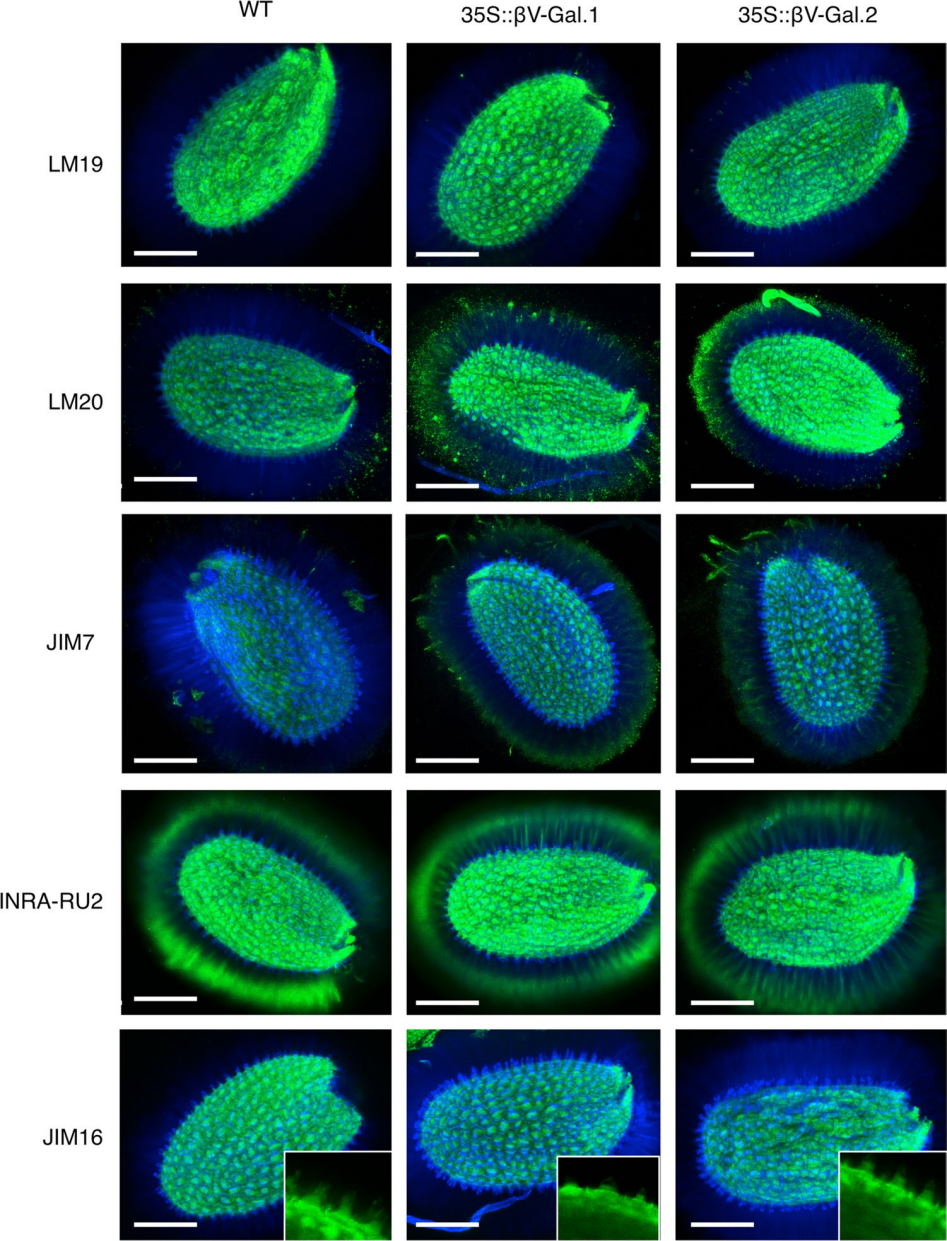
Confocal images (maximun projections) showing WT, 35S::βV-Gal.1 and 35S::βV-Gal.2 seeds immunolabelled with antibodies against non methyl esterified HG (LM19), methyl esterified HG (LM20/JIM7), RGI (INRA-RU2), and AGII (JIM16). All images show counterstain with calcofluor (blue fluorescence) except magnifications of JIM16 labelling. Scale bars = 200 µm

Immunolocalization studies (Fig. 5) confirm the absence of changes in the epitope recognized by LM19 antibody as well as the increase in the signal corresponding to methyl esterified HG (LM20) in transgenic 35S::βV-Gal seeds, especially in the outer region of adherent mucilage, but also in the columella and in seed coat (Fig. 5, quantified in Supplementary Fig. S8). The same labelling pattern, although with lower intensity is observed with JIM7 antibody. Regarding RGI, we can observe changes in labelling distribution in 35S::βV-Gal seeds, with a higher labelling with INRA-RU2 antibody through the rays conforming adherent mucilage (Fig. 5), although the absence of changes in the ELISA experiments point to an increase of the exposure of this epitope rather than an increase in the levels of RGI. It should be noted that, of the two anti AGII antibodies used, signal could only be detected, at a very low level, with JIM16 in the seed coat columella. As expected, the signal is reduced in both transgenic lines (magnification in Fig. 5).

### Changes in 35S::βV-Gal seed coat mucilage pectins have not compromised the correct hydration of the seeds

**Fig. 6:**
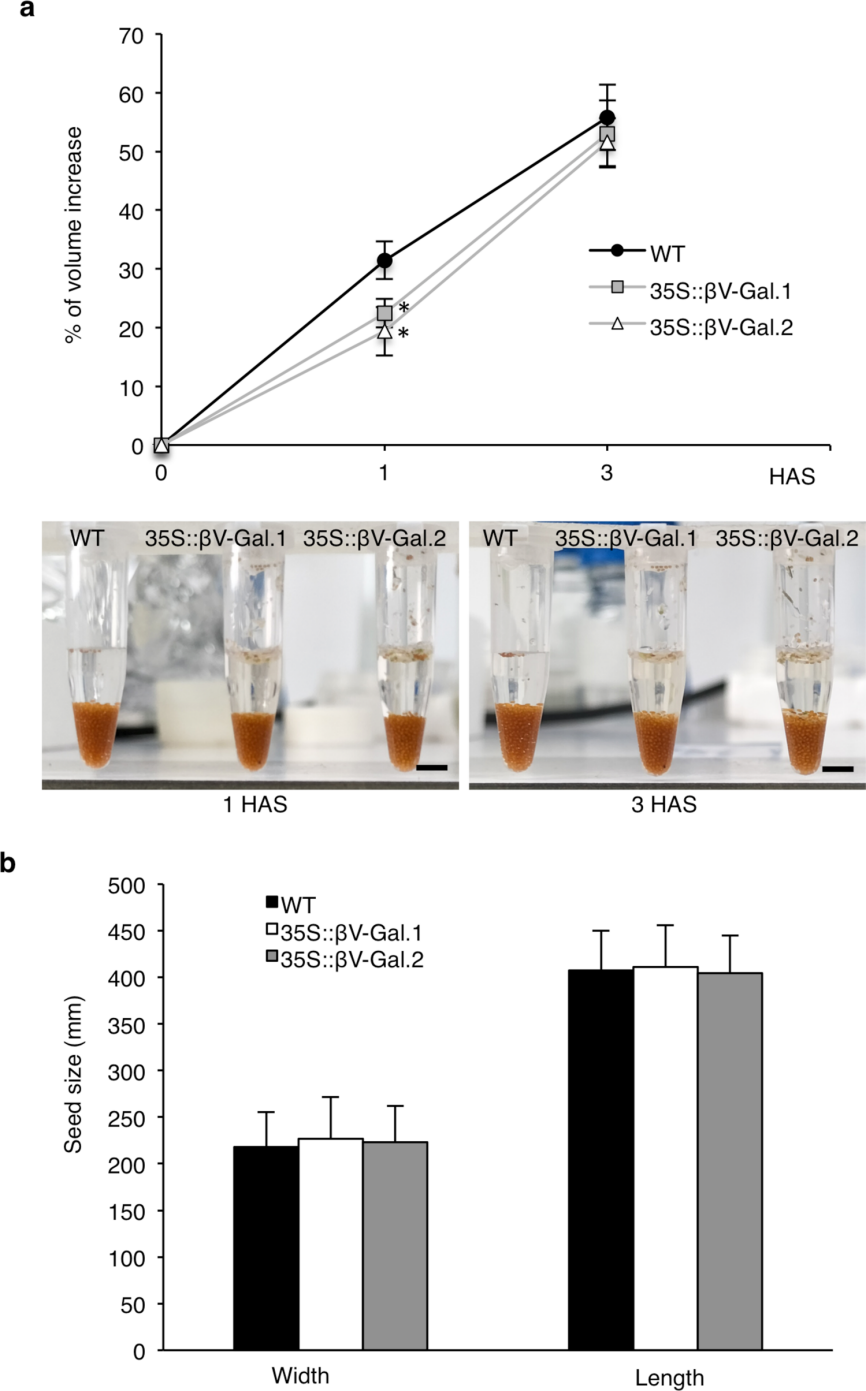
**a** Water absorption capability of WT, 35S::βV-Gal.1 and 35S::βV-Gal.2 seeds. Data are expressed as percentage of volume increase of the seeds in the tubes after soaking in water for 1 and 3 h after soaking (HAS). An example photograph of each tube is included below the graph. Scale bars = 0.5 cm. **b** Size (width and length) of dry WT, 35S::βV-Gal.1 and 35S::βV-Gal.2 seeds. Values are the means of three biological replicates ± SD. Asterisks indicate the level of significance (Student’s t test): *p < 0.05

Given the implication of seed mucilage in seed hydration, we tested whether changes in its pectic component result in an alteration of this process. To do so, we weighed the same amount of dry WT and 35S::βV-Gal seeds and incubated each batch of seeds in water at room temperature to determine changes in the volume of the hydrated seeds. Despite the above-mentioned changes in the mucilage of the transgenic lines, seed hydration has not been seriously compromised. Only a slight delay is observed in the initial stages of this process, although at three hours after imbibition the hydration levels are similar in the transgenic seeds and the WT (Fig. 6a). This slight difference does not arise from altered seed size, since no significant differences between transgenic lines and wild-type width or length have been detected (Fig. 6b), and it does not compromise seed germination.

## Discussion

The main objective of this work was to determine the role played by AGII of AGPs, and more specifically, the galactose residues of its side chains, in the structural organization of the cell wall. This was achieved by studying the alterations caused *in muro* by the reduction of these galactose substitutions. We decided to focus our work on the characterization of seed coat mucilage, not only because of the role that AGPs seem to play in its proper deposition and secretion, but also because of the growing interest that this material is gaining as a model system for the study of cell wall structure (Griffiths et al. 2016). For this purpose, we have used *Arabidopsis thaliana* plants that overproduce the β-galactosidase βV-Gal from *Cicer arietinum* (35S::βV-Gal.1 and 35S::βV-Gal.2 plants).

The βV-Gal enzyme transiently overproduced in *Nicotiana benthamina* leaves acts specifically on β-(1,3) and β-(1,6)-galactosyl bonds of poly and oligosaccharides (with a slightly higher activity against the latter), including commercial gum arabic and synthetic β-(1,3) (1,6)-galactan (Tables 1, 2). These results point out that it may act on AGII of AGPs, similarly to β-galactosidases from other species such as radish or rice (Kotake et al. 2005; Hoang et al. 2023). This fact was confirmed in *A. thaliana* plants overproducing the βV-Gal enzyme by means of ELISA experiments with JIM14 and JIM16 antibodies, specific for galactose residues of AGII, which we tested against seed protein extracts (Fig. 1a) and seed mucilage (Fig. 1b, c). These results prove the action of chickpea βV-Gal on *A. thaliana* AGPs, which in the case of the seed mucilage, were only detected in the CDTA- and KOH- extracted fractions. The reduced staining intensity with ruthenium red (a dye with high affinity for pectins) in the adherent mucilage of the transgenic seeds compared to WT (Fig. 2a, b and S3) evidenced the relationship of AGII (and consequently AGPs) and the seed mucilage. This suggests that the previously observed reduction of the galactose chains of AGII, could have led to reduced mucilage adhesion to the seed coat or defects in its secretion in the 35S::βV-Gal seeds.

Arabidopsis mutants lacking various glycosyl-transferases involved in AGII synthesis, such as galactosyl-transferases or glucuronosyl-transferases, secrete a lower amount of mucilage after hydration (Basu et al. 2015; Showalter and Basu 2016). However, when we quantify the sugars present in both adherent and non-adherent mucilage of 35S::βV-Gal and WT plants, we observe just a slight reduction in transgenic seeds (in total sugars and uronic acids), which is only statistically significant in the case of non-adherent mucilage (Table 3). This could suggest that the modifications in AGII galactose residues produced by βV-Gal are not sufficient to cause a more apparent loss of mucilage. In fact, significant reductions in mucilage in plants deficient in AGII synthesis are usually achieved when several isoforms of glycosyltransferases are mutated simultaneously (Basu et al. 2015). Therefore, the lower intensity of mucilage staining with ruthenium red (Fig. 2) might be due to changes in mucilage composition or in the distribution of its components, rather than to a reduction in the amount of the mucilage secreted. Similarly, the *sos5* mutant of Arabidopsis (knockout mutant for the AGP SALT-OVERLY SENSITIVE 5) showed a higher proportion of galacturonic acid in the non-adherent mucilage with respect to the adherent mucilage, without changing its total amount (Griffiths et al. 2014).

The ELISA analysis of non-adherent mucilage (extracted with H_2_O) showed no differences between WT and 35S::βV-Gal plants in any of the pectic or hemicellulosic polysaccharides levels (Supplementary Fig. S5), which a priori would indicate that in our case there is no increase in the extractability of any of the analysed polymers. However, differences are observed in the pectin-rich fraction of adherent mucilage (extracted with CDTA), where the epitopes corresponding to methyl esterified HG, recognized by JIM7 (partially methyl esterified HG) and LM20 (HG with high degree of methyl esterification), show a remarkably higher signal in 35S::βV-Gal seeds than in WT seeds (Fig. 3a). This increase in the level of HG methyl esterification explains the lower intensity observed in the staining with ruthenium red in the transgenic seeds (Fig. 2a, b and S3), as this dye has an affinity for the free carboxyl groups of pectins (Sterling 1970).

The fact that no differences were observed in the levels of un-methyl esterified HG (LM19) in the H_2_O and CDTA fractions (Fig. S5a and 3a) and that the amount of uronic acids in these fractions show only minor changes (Table 3), suggests that the variations in LM20 and JIM7 epitopes are due to an increase in the methyl esterification degree (as confirmed by increased methyl content in CDTA-extracted mucilage shown in Fig. 4a and the decrease in PME activity in Fig. 4b) and neither the overall amount of HG or its distribution in the different mucilage layers are affected. This is supported by the absence of notable changes between transgenic and WT seeds in the levels of uronic acids or LM19 signal in the fraction extracted with KOH (Table 3 and Supplementary Fig. S6).

Notably, despite being the fraction enriched in hemicelluloses, considerably high levels of RGI are detected, which could imply a direct interaction between this polymer and hemicellulosic polysaccharides, like xylan or XG, as described in other tissues, such as Arabidopsis stems or roots (Cornuault et al. 2014). Besides, AGII from AGPs has even been proposed as the link between pectin and hemicelluloses in different tissues, like Arabidopsis cell cultures or Siberian fir and Norway spruce foliage (Tan et al. 2013; Makarova and Shakhmatov 2021; Shakhmatov and Makarova 2024). However, our results do not support this role of AGII in seed mucilage, given the absence of changes in the relative levels of these polysaccharides through the different mucilage layers in 35S::βV-Gal seeds, or at least indicate that the complete integrity of AGII galactose residues is not essential for this purpose.

It is widely known that the degree of esterification of pectins strongly controls the mechanical properties of the cell wall, determining its stiffness, charge and even its hydration (Willats et al. 2001b). Even though HG is not as quantitatively important as RGI, seed mucilage expansion and secretion are greatly influenced by HG esterification. Different mutants with reduced levels of HG methylation (either by reduced methyltransferase activity or by mutations in PMEs inhibitors) or with increased methylation levels, by loss of PMEs activity, present severe alterations in mucilage hydration capacity, changes in its adherence and failures in its secretion (Du et al. 2022; Saez-Aguayo et al. 2013; Voiniciuc et al. 2013; Turbant et al. 2016). Similarly, mutants in *FLY1* (encoding FLYING SAUCER1, a transmembrane E3 ubiquitin ligase) with reduced degree of pectin methyl esterification, show increased mucilage adherence and defective mucilage extrusion (Voiniciuc et al. 2013; Kunieda et al. 2020).

These defects in mucilage secretion and adherence are also observed when the correct synthesis of AGII side chains is impaired (Showalter and Basu 2016) or the metabolism of neutral RGI side chains is altered. For instance, when the gene encoding the β-galactosidase MUM2 of Arabidopsis, which acts on the galactan chains of RGI, is mutated (Dean et al. 2007). Also, mutations of the Arabidopsis UDP-rhamnose/galactose transporter 2 (URGT2), which cause a reduction in RG-I content, induce changes in mucilage composition and structure, including alterations of HG methyl esterification (Parra-Rojas et al. 2019). All these studies indicates that a balance between the different components of the mucilage is essential, and that precise regulation of their synthesis is necessary for their proper interactions and for their correct deposition and extrusion, as evidenced by our results. In addition, previous studies have already proposed a link between AGPs and HG methylation in Arabidopsis seed coat mucilage. In this regard, it has been reported that SOS5, an AGP required for mucilage adherence, suppresses the previously mentioned *fly1* phenotype (Griffiths et al. 2016) and mutations in the Golgi S-adenosylmethionine (SAM) transporters GoSAMT1 and GoSAMT2, involved in mucilage HG methyl esterification, result in increased levels of AGPs (Parra-Rojas et al. 2023).

In our case, the increase in the HG methylation degree appears to be a compensatory mechanism to maintain the mechanical properties of the mucilage, in response to the βV-Gal-mediated reduction of AGII neutral side chains of AGPs. The neutral side chains of RGI have been described to control the degree of cell wall porosity and proposed to keep adjacent HG domains apart (Carpita and Gibeaut 1993; Harholt et al. 2010), preventing their cross-linking by calcium bridges, that occur in the absence of methyl esterifications (Willats et al. 2001b). Considering our results, AGII side chains of AGPs could be performing a similar function during mucilage deposition and their reduction could be triggering the increase in methyl esterification degree, probably during the formation of this structure, as deduced by reduced PME activity detected in protein extracts form dry 35S::βV-Gal seeds when compared to the WT shown in Fig. 4b. The fact that 35S::βV-Gal seeds do not exhibit drastic changes in the extractability of the different polymers, nor in the relative amounts of non-adherent/adherent mucilage, could be determined by this homeostasis mechanism. The increase in esterification would prevent a possible increase in HG-chain interactions resulting from the reduction of AGII chains, which could otherwise lead to a redistribution of polysaccharides between the different layers, and explain the lack of aberrant mucilage structures, defects in its extrusion or even in its detachment from of the primary walls of the seed coat epidermal cell (Figs. 2 and 5) as seen in different mucilage mutants with incorrect HG interactions (Saez-Aguayo et al. 2013; Kunieda et al. 2020). We have observed only a slight decrease in the amount of total sugars (Table 3), which, especially in the case of non-adherent mucilage, could be attributed to the increase in methyl esterification in the outermost layers of adherent mucilage (Fig. 5), that could be causing a loss of interactions between polysaccharides of the different layers. These changes have not compromised the correct hydration of the seeds (Fig. 6), despite the known implications of methylation degree in this process (Ajayi et al. 2021; Saez-Aguayo et al. 2013). In our case, this increase in pectin methylation has only produced a slight delay in seed hydration, which confirms the role of HG methylation in water adsorption capacity, but may also suggest that the esterification pattern itself, rather than the total amount of methyl groups, influence this process, as proposed earlier (Willats et al. 2001b).

In any case, our work shows that galactose residues of AGII present in AGPs play a role in maintaining the properties of Arabidopsis seed coat mucilage, and its modification in *muro* is sufficient to trigger a compensatory mechanism to maintain mucilage homeostasis and the correct interactions of the polysaccharide components of this structure through the control of HG methyl esterification degree, which is a key factor in determining the mechanical properties of this structure. The signaling mechanisms involved in this process remain to be determined, but according to our results, glycosylation of AGPs may be a key point, as suggested earlier (Basu et al. 2015), and they represent a further step in completing the entire picture of the mechanisms that control the correct synthesis, secretion and expansion of this structure. Furthermore, considering the interest of mucilage as a model for the study of the cell wall, the 35S::βV-Gal plants developed for this study provide a good system for determining the role of AGPs in cell wall metabolism during other developmental processes.

## Supplementary Information

Below is the link to the electronic supplementary material.

Supplementary file1 (PDF 1446 KB)

## Data Availability

All generated data are included in this article.
