# Supplementary material for: Reduction of galactose side chains in type II arabinogalactan alters homogalacturonan methyl esterification in Arabidopsis thaliana seed coat mucilage
Source: Planta. 2025 May 24;262(1):4. doi: 10.1007/s00425-025-04717-x (PMC12103386; doi:10.1007/s00425-025-04717-x)
Supplement: Supplementary file 1 — Supplementary file1 (PDF 1446 KB) [file 425_2025_4717_MOESM1_ESM.pdf]

Supplementary Table S1 Primer sequences used in this work. *attB* sites are underlined

Target	Purpose	Primer sequence 5'-3'
CarBGal5	Cloning	GGGGACAAGTTTGTACAAAAAAGCAGGCTGCATGGAAACCAACTCAGTTTCC<br>GGGGACCACTTTGTACAAGAAAAGCTGGGTCTTATACTTGTGTTGTATTAGTTC
	Sequencing/expression analysis (CarBGal5-Fwd)	GCATTCGCAGTTGCTCG
	Sequencing/expression analysis (CarBGal5-Rev)	GTCGAGACCATGGAGCA
	Sequencing	GACCTTATGTTTGTGCTGA
	Sequencing	GTTCACTTGATGCTGGAG
attL1	Sequencing	TCGCGTTAACGCTAGCATGGATCTC
attL2	Sequencing	GTAACATCAGAGATTTTGAGACA
ACT2	Expression analysis in seedlings (ACT2-Fwd1)	CTCCCGCTATGTATGTCGCC
	Expression analysis in seeds (ACT2-Fwd2)	CACCCTGTTCTTCTTACCGAGGC
	Expression analysis in seedlings/seeds (ACT2-Rev)	TTGGCACAGTGTGAGACACAC

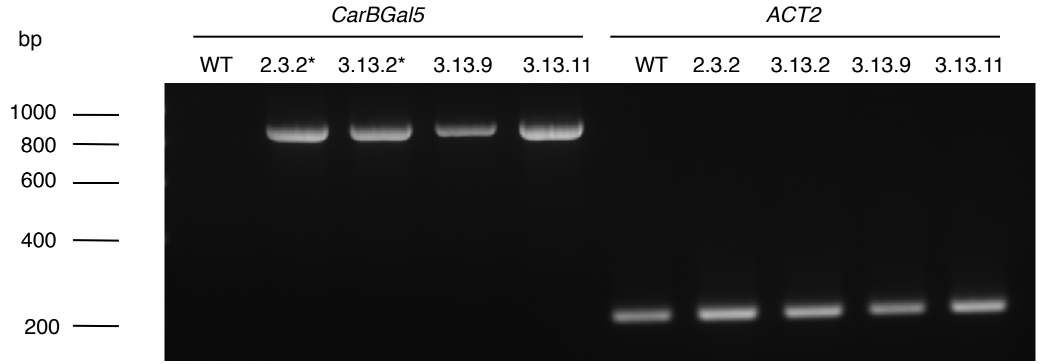

Supplementary Fig. S1 Transgenic 35S::βV-Gal line selection. Agarose gel electrophoresis of the PCR products obtained after amplification of *CarBGal5* and *ACT2* cDNA from WT and 35S::βV-Gal 10-day-old whole seedlings. Numbers indicate different 35S::βV-Gal lines shown as example. Stars marks the selected lines. The primer pairs used for expression analyses were CarBGal5-Fwd and CarBGal5-Rev (amplicon size of 898 bp) and ACT2-Fwd2 and ACT2- Rev (amplicon size of 200 bp)

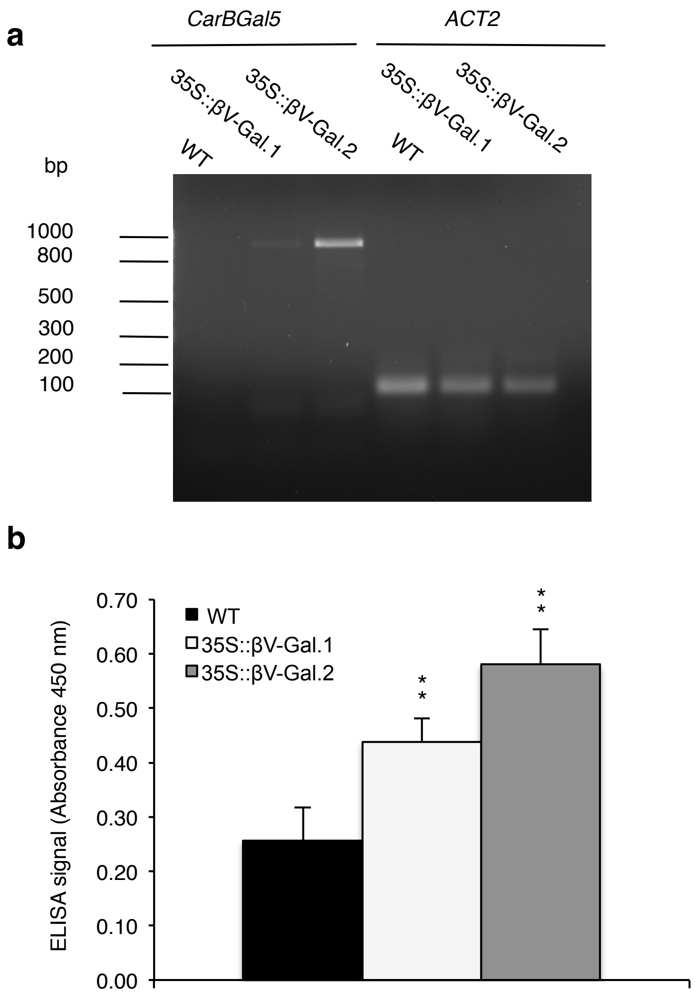

Supplementary Fig. S2 Analysis of the presence of *CarBGal5* transcripts and βV-Gal protein in transgenic seeds. **a** Agarose gel electrophoresis of the PCR products obtained after amplification of *CarBGal5* and *ACT2* cDNA from WT, 35S::βV-Gal.1 and 35S::βV-Gal.2 seeds. 0.01. The primer pairs used were analyses were CarBGal5-Fwd and CarBGal5-Rev (amplicon size of 898 bp) and ACT2-Fwd1 and ACT2-Rev (amplicon size of 115 bp). **b** ELISA analysis of protein extracts from WT, 35S::βV-Gal.1 and 35S::βV-Gal.2 with anti-βV-Gal antibodies. Values are the means of three biological replicates ± SD. Asterisks indicate the level of significance (Student's t test): $^{**}p < 0.01$

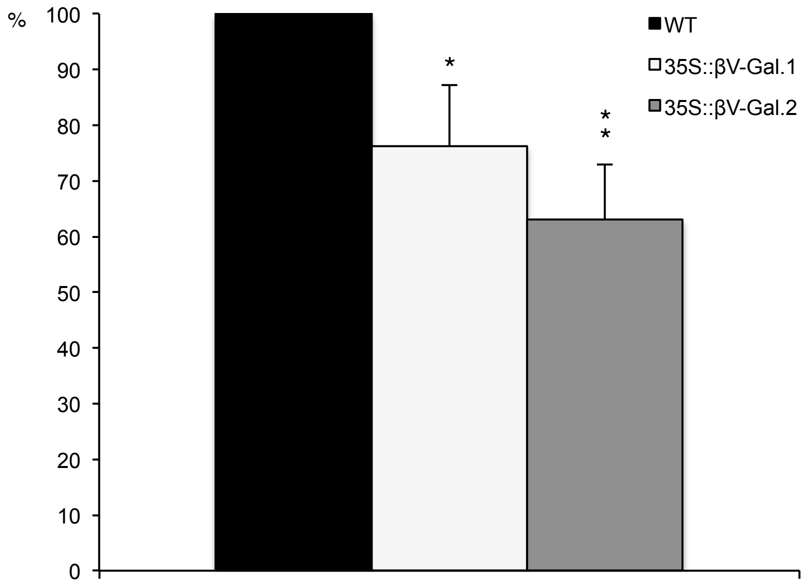

Supplementary Fig. S3. Quantification of ruthenium red staining of WT, 35S::βV-Gal.1 and 35S::βV-Gal.2 seeds shown in Fig. 1. Results are expressed as a percentage of variation in transgenic lines with respect to the WT, to which a value of 100% is assigned. Values are the means of six biological replicates ± SD. Asterisks indicate the level of significance (Student's t test): *p<0.05; **p < 0.01

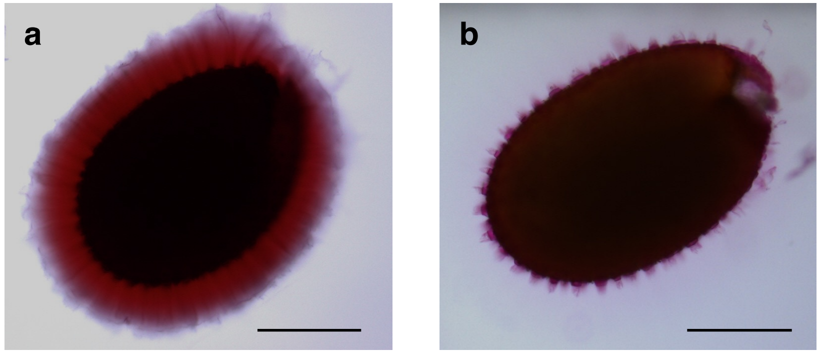

Supplementary Fig. S4 *A. thaliana* WT seeds stained with ruthenium red after mucilage extraction with H_2O (a) and CDTA (b) for 30 minutes at 250 rpm. Scale bars = 200 µm

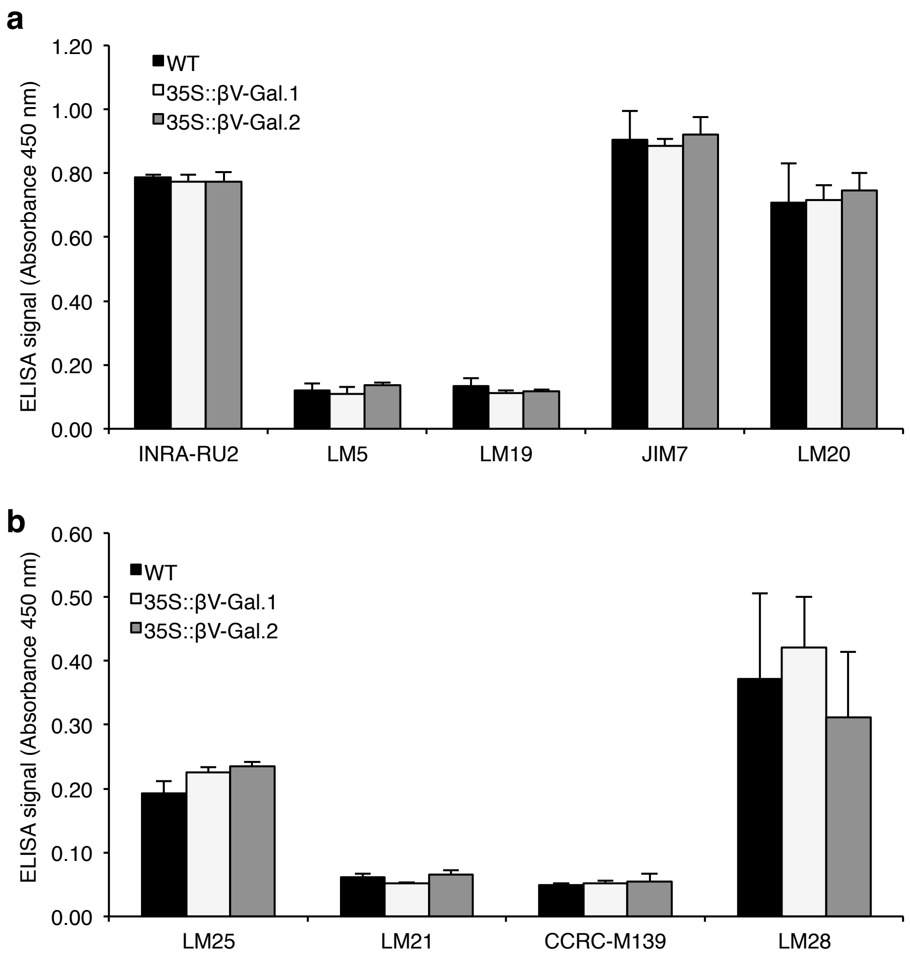

Supplementary Fig. S5 ELISA analysis of non-adherent mucilage extracted with H_2O from WT, 35S::βV-Gal.1 and 35S::βV-Gal.2 seeds. **a** ELISA signal for antibodies against pectic polysaccharides RGI (INRA-RU2), β-D-(1,4)-galactan (LM5) and HG (non methyl esterified: LM19, partially methyl esterified: JIM7, methyl esterified: LM20). **b** ELISA signal for antibodies against the hemicelluloses XG (LM25), β-(1,4)-mannan oligosaccharides (LM21), xylan/heteroxylan (CCRC-M139) and glucuronoxylan (LM28). Values are the means of three biological replicates ± SD. Signal intensity should not be compared between different antibodies as their epitope binding affinities can vary

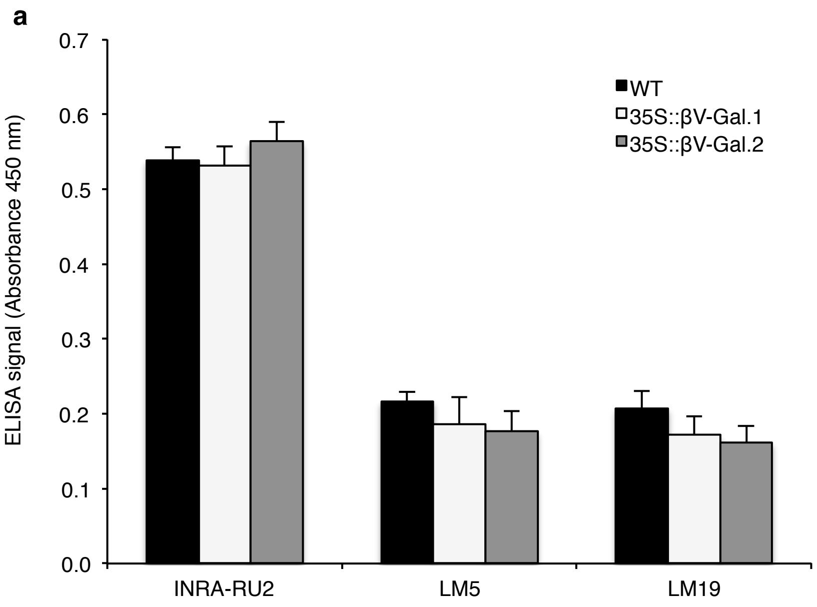

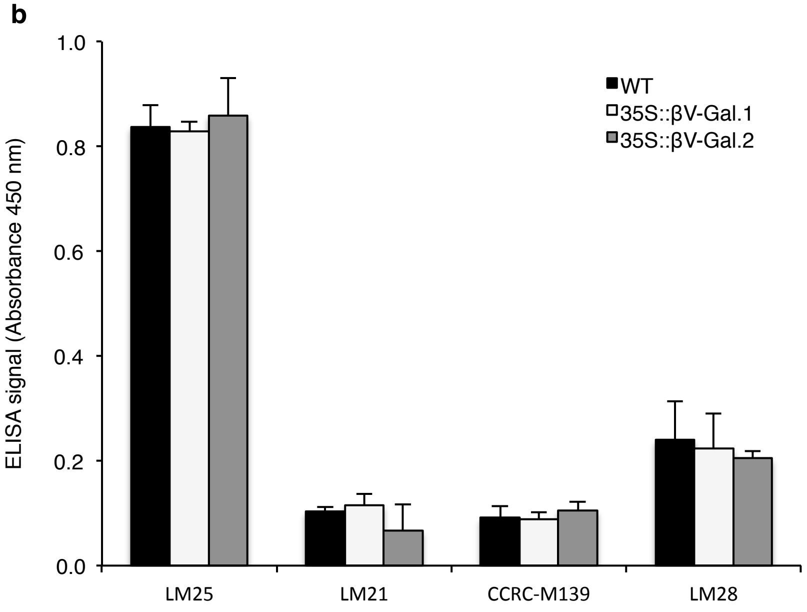

Supplementary Fig. S6 ELISA analysis of adherent mucilage extracted with KOH from WT, 35S::βV-Gal.1 and 35S::βV-Gal.2 seeds. **a** ELISA signal for antibodies against pectic polysaccharides RGI (INRA-RU2), β-D-(1,4)-galactan (LM5) and HG (non methyl esterified: LM19, partially methyl esterified: JIM7, methyl esterified: LM20). **b** ELISA signal for antibodies against the hemicelluloses XG (LM25), β-(1,4)-mannan oligosaccharides (LM21), xylan/heteroxylan (CCRC-M139) and glucuronoxylan (LM28). Values are the means of three biological replicates ± SD. Signal intensity should not be compared between different antibodies as their epitope binding affinities can vary

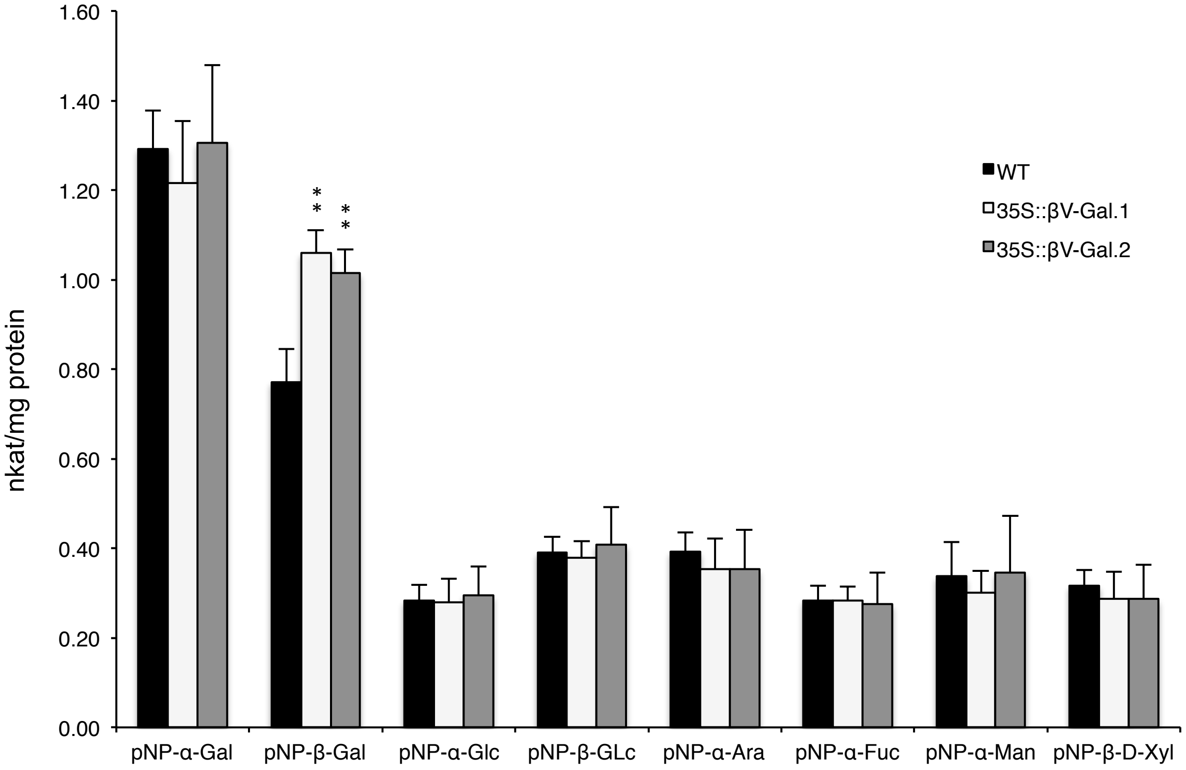

Supplementary Fig. S7 Activity of WT, 35S::βV-Gal.1 and 35S::βV-Gal.2 seed protein extracts toward pNP substrates. Data are expressed as nkat/mg protein. Values are the means of three biological replicates ± SD. Asterisks indicate the level of significance (Student's t test): **$p < 0.01$

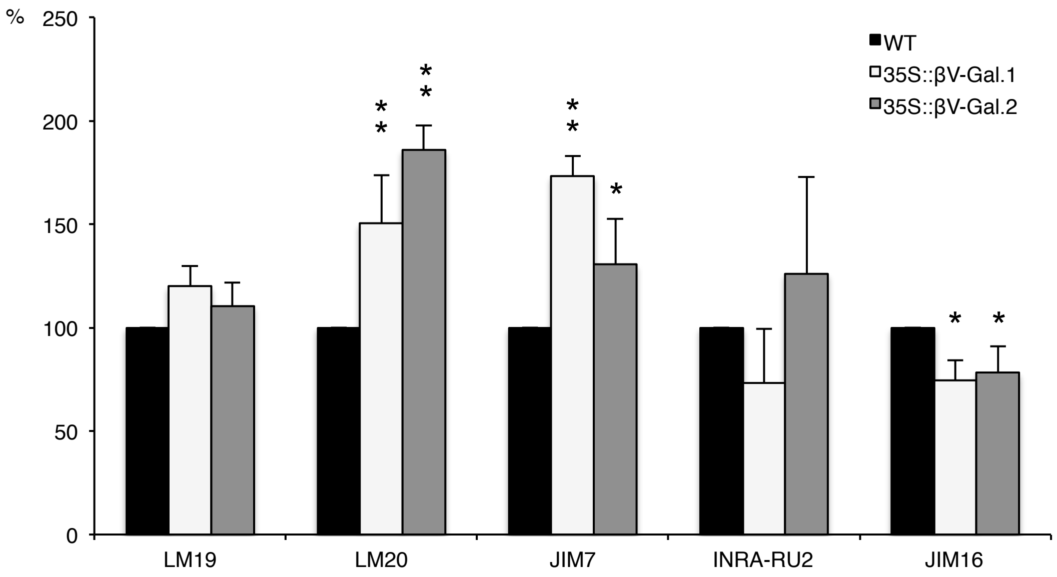

Supplementary Fig. S8 Fluorescence quantification of confocal images (maximum projections of FITC channel) from immunolabelling experiments of WT, 35S::βV-Gal.1 and 35S::βV-Gal.2 seeds with antibodies against non methyl esterified HG (LM19), methyl esterified HG (LM20/JIM7), RGI (INRA-RU2), and AGII (JIM16). Example images are shown in Fig. 5. Results are expressed as a percentage of variation in transgenic lines with respect to the WT, to which a value of 100% is assigned. Values are the means of three biological replicates ± SD. Asterisks indicate the level of significance (Student's t test): *p<0.05; **p < 0.01
